# The Ability of Vibrational Spectroscopy to Analyze Holistically the Food Matrix-Moving Away from the Concept of Individual Compounds

**DOI:** 10.3390/mps9020058

**Published:** 2026-04-02

**Authors:** Daniel Cozzolino

**Affiliations:** Centre for Nutrition and Food Sciences, Queensland Alliance for Agriculture and Food Innovation (QAAFI), The University of Queensland, Brisbane, QLD 4072, Australia; d.cozzolino@uq.edu.au

**Keywords:** food, matrix, ingredients, spectroscopy, fingerprinting, nutrition

## Abstract

The concepts of food matrix and holistic analysis have been used in a wide range of scientific disciplines to describe the sum of the parts of a whole that provide a specific property or functionality to the sample. Traditional chemical and physical analysis needs to destroy the sample (e.g., dilution, extraction, drying) before analysis. The utilization of vibrational spectroscopy techniques, like near (NIR), mid infrared (MIR) and Raman spectroscopy, allows for the non-destructive analysis of food ingredients and products. The resulting output of this analysis is based on the information provided by the vibrational modes of atoms present in the different molecules, allowing the measurement of different chemical and physical characteristics of the food. The objective of this paper is to discuss the ability of vibrational spectroscopy methods to provide robust tools to analyze the food matrix holistically, moving away from the traditional analysis of individual compounds or chemical parameters. Studies discussed and presented in this review demonstrated the ability of vibrational spectroscopy (e.g., NIR, MIR and Raman spectroscopy, hyperspectral imaging) to assess the whole food matrix beyond the traditional notion of developing a calibration model.

## 1. Introduction

The definition of matrix is associated with “*something where other things are embedded*” [1,2,3]. This definition has been used in a wide range of scientific disciplines to better understand and describe those parts of a whole that provide either specific properties or functionalities to the sample [2]. It is well known that advances in both food chemistry and nutrition have contributed to the decrease in nutrient deficiencies by advocating for the consumption of the required quantity of nutrients through the ingestion of food ingredients or supplements (e.g., scurvy and ascorbic acid, pellagra and niacin, beriberi and thiamin, rickets and vitamin D, and neural tube defects and folic acid) [1,2,3,4].

Similarly, the practice of nutrition has emphasized the chemical composition and nutritional content of food ingredients or products in the so called “*nutritionism*” [1,2]. However, this approach has been challenged by the fact that several nutrients do not perform equally when studied in isolation, or independently of their interactions within the whole food matrix or meal [1,2]. It has been determined that both food ingredients and products with identical chemical composition determine differences in the amount and type of nutrient supplied as well as their biological function in the human (e.g., metabolism) [3]. These effects might influence, among others, the integrity and diversity of gut microbiota, determining differences in the health and nutritional outcomes for humans [1,2].

Differences in either the functionality or nutritional value in both food ingredients and products are also dependent on different interactions that occur between compounds and nutrients present in a sample, either positively (even synergistic) or negatively [5,6,7,8]. Furthermore, the interactions that occur in the food product or meal, and between the different food ingredients that are part of the whole meal, will have an effect in the nutrition and health of the individual [5,6,7,8].

**Figure 1:**
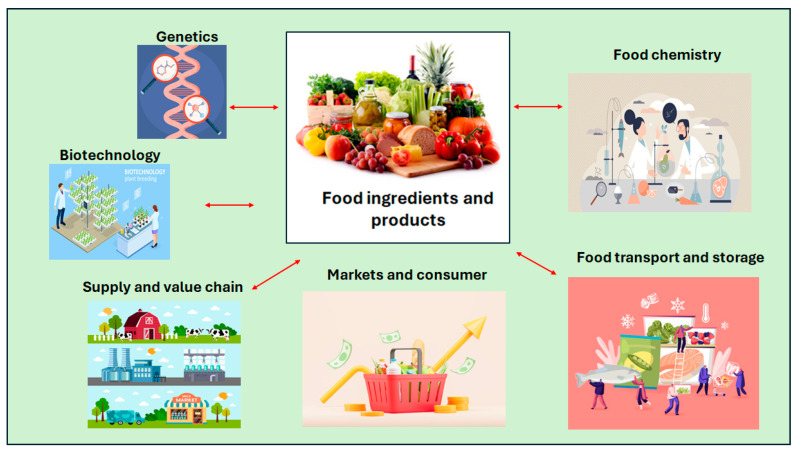
The complexity of food—the different interactions between chemistry, physics and other fields that influence the whole food (e.g., ingredients or products).

It is also known that a high amount of a given nutrient (e.g., vitamins and antioxidants) usually does not have nutritional or health benefits and may cause some deleterious effects in some individuals [9]. Yet, the current approach in food chemistry and nutrition, is still focused on the evaluation or analysis of a “*single or isolated nutrient*”. This approach is still driving research and development on the effects on nutrition and health; however, in some cases it produces conflicting results which are difficult to interpret [1,4]. Nevertheless, food is complex, where variables such as the production system, genetics, processing methods (e.g., milling, extrusion), storage conditions (e.g., temperature, time), and transport, among other factors or variables, modulate the variability in the chemical and other characteristics of food ingredients or products. This complexity is made up by the concentration of different compounds, the interactions between these chemical compounds with physical characteristics or properties of the sample. It is this complexity, rather than individual compounds that determine the functionality and nutrition value of food ingredients and products. Figure 1 illustrates the complexity of food and the different interrelationships with different components of the food system.

Currently, there is an increasing interest in moving from the analysis of either single compounds or nutrients (reductionist approach) to a more holistic understanding of the food matrix (e.g., chemistry, physical properties, functionality) [10,11]. This line of thinking is gaining particularly traction in both food chemistry and nutrition research [10,11,12,13,14]. However, one of the bottle necks of this approach is the limitations of the current analytical methods and techniques that are utilized to evaluate the whole food matrix [12,13,14].

The objective of this paper is to discuss the ability of the different vibrational spectroscopy methods to provide robust tools to analyze the food matrix holistically, moving away from the traditional focus on the analysis of individual compounds or chemical parameters (calibration development).

## 2. Scope of This Review

This review intends to critically discuss the utilization of vibrational spectroscopy to analyze the whole food matrix. Vibrational spectroscopy and related methods such as near-infrared (NIR), mid infrared (MIR) and Raman spectroscopy are well established analytical methods that allow us to measure the chemical composition and nutritive value of food ingredients and products. However, the analytical information obtained by these methods is not fully utilized or interpreted. This review does not repeat the content of previous papers published in the scientific literature, dedicated to reporting applications of these techniques to predict the chemical composition and nutritive value of different foods or to target issues associated with food authenticity, fraud. It is also beyond the scope of this paper to discuss the advantages and limitations of the different chemometric or machine learning (ML) tools used to develop a given application.

## 3. Single Components, Ingredients and Molecules vs. The Matrix

The European Food Safety Authority (EFSA) defines “*functional food*” as the “*food or food ingredients that determines an effect in one or more target functions in the whole body, beyond adequate nutritional effects, in a way that is relevant to either an improved state of health and well-being and/or reduction in risk of disease*” [15]. This definition emphasizes that it is the food matrix and not a single compound or food property that determines its functionality [15]. Thus, the functional and nutritional properties of food ingredients and products are evaluated using different analytical methods and techniques based on well-known physico-chemical principles [8,16,17,18,19]. However, the utilization of these analytical methods targets one compound, component, or property to explain the functionality of the food matrix [10,11,12,13,14]. Consequently, the analyses of single and different compounds and molecules in the food (e.g., proteins, starch, bioactive compounds) are used to estimate their functionality or nutritional value. These single chemical compounds are used as proxies to infer their effects on physiological processes that contribute to maintaining human nutrition and health status [10,11,15].

Most of the current chemical and physical analytical methods (e.g., proximate analysis, instrumental methods) utilized to evaluate and measure food chemical composition, physical properties and functionality are destructive and time consuming [20,21,22,23]. During the implementation of these methods, the sample is required to be destroyed before analysis. In this process, the sample is handled by cutting, grinding, or homogenizing using different techniques. The sample is also dried (e.g., oven drying, freeze drying), compounds extracted using different solvents, or diluted, before weighted and introduced into the state-of-the-art instrumentation to be analyzed [24,25,26,27]. After following different steps during the analytical process, a chemical compound or property is therefore measured and used to characterize the whole food ingredient or product [24,25,26,27]. As stated by different authors, the current analytical methods utilized in food chemical analysis have become a leftover of a past era that is still based on fragmentary, brutal chemical techniques reporting results too late to be of any use in decision-making [24,25,26,27]. Even more importantly, research must accept restrictions on the range of analysis or tests as well as the number of samples that can be afforded to be tested due to the analytical cost of the current analytical methods [24,25,26,27].

**Figure 2:**
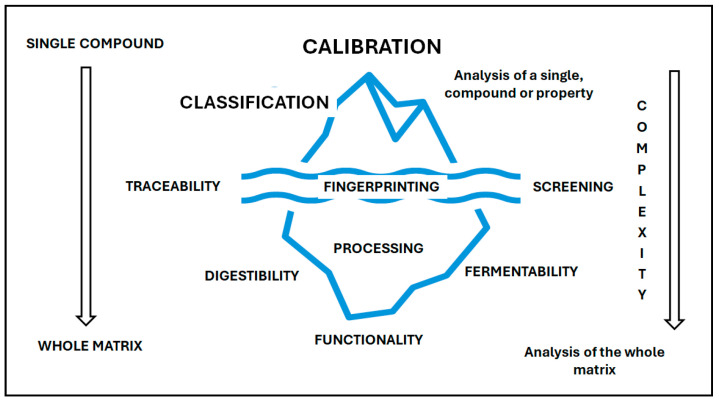
The utilization of vibrational spectroscopy to analyze the food matrix will improve our understanding about food ingredients and products, moving away from the routine analysis of single compounds—known as calibration.

Vibrational spectroscopy techniques have demonstrated their role as analytical methods to analyze food ingredients and products in different applications [27]. Most of these techniques are characterized by their non-destructive nature allowing for the analysis of whole grains, intact muscles, etc. The analysis of samples using these techniques is based on the collection and interpretation of the information that originated from the different vibrational modes of atoms in the molecules present in a sample. This information allows the measurement of the different properties (e.g., chemical composition, physical changes) of the sample [28,29,30,31,32]. In this way, a wide range of compounds, bioactive molecules (e.g., volatile and phenolic compounds), and macro and micro elements have been either measured or predicted in different food ingredients and products using techniques such as MIR, NIR and Raman spectroscopy [28,29,30,31,32,33,34]. The best-known application of vibrational spectroscopy techniques is through the process of predicting or measuring a compound or chemical property—called calibration. However, the utilization of these techniques to analyze the food matrix will improve our understanding about food ingredients and products, providing tools that will allow us to move away from the solely analysis of single compounds—calibration (Figure 2). As shown in Figure 2, the utilization of vibrational spectroscopy can be viewed as an iceberg. The development of calibration, and to a lesser extent the use of classification (e.g., authentication, fraud), are the most widely known applications of these techniques. However, the use of spectra to understand the whole food matrix or even its use to better understand food functionality, food–nutrition interactions still lay under the surface. The arrows indicate the direction of movement from the development of calibration to a more holistic analysis. The complexity increases from the single compound to the analysis of the whole food matrix.

**Figure 3:**
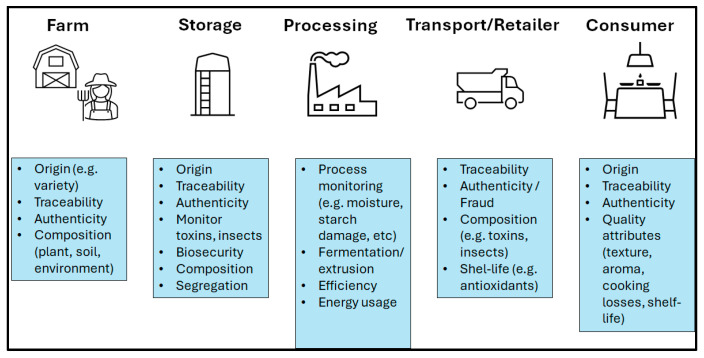
The food matrix rather than the analysis of individual or single compounds is the focus of the analysis, with the goal to either assess or better manage the different steps of the food supply and value chain—from farm to fork.

As stated in the above section, the vibrations by the molecules originated in the electromagnetic spectrum not only allow us to predict the chemical composition of the samples but also to infer the functional properties of the food [27]. This can be achieved by comparing the spectrum of the unknown sample with data libraries of spectra combined with reference samples (e.g., in house library of food ingredients). The spectrum might be analyzed directly by interpreting both the position and intensity of peaks or absorbances at specific frequencies or wavenumbers [28,29,30,31,32,33,34]. A myriad of studies have reported the ability of vibrational spectroscopy (e.g., NIR, MIR, Raman spectroscopy) to assess the functionality of different food ingredients and products. In these types of applications, the spectrum of the sample represents the whole makeup of the sample rather than the sum of the single components presented in the individual food sample. Consequently, the ability to analyze the matrix has become of great interest to better understand, control or even manage the complexity of food and the different issues and interactions that emerge along the supply and value chain. This approach becomes even more important in the fork-to-farm or product-to-ingredient strategies defined in different countries or regions (European Community, 2020) [35]. Figure 3 illustrates the different steps in the food value chain (from farm to fork) where this approach has been evaluated and implemented by researchers and the food manufacturing industry. The analysis of a whole commodity or food ingredient rather than the analysis of individual or single compounds is the focus of the application. The assessment and management of the different steps of the food supply and value chain—from farm to fork has been possible by the utilization of vibrational spectroscopy.

## 4. The Analysis of the Food Matrix—Examples

### 4.1. Analyzing the Structures That Makes up Food

The scientific literature in the field of vibrational spectroscopy provides examples of the utilization of these techniques to analyze the whole food matrix [29,36,37,38,39]. The utilization of both MIR and Raman spectroscopy was reported to evaluate the molecular and crystal lattice vibrations, and their relationships with the identification of different crystalline forms (A- and B-forms) and protein structure in different active compounds and food products [36,37,38,39]. The identification of amorphous lactose powder from the crystalline lactose was possible by the different numbers of peaks as well as the less defined peaks of the amorphous lactose spectrum [29,36,37,38,39,40]. Vibrational spectroscopy has also been reported to assess the structure of proteins, where specific frequencies in the MIR spectral region are known to be associated with both the amide I and amide II groups [39]. Different food proteins have a unique structure that can be identified by their distinctive pattern in hydrogen bonding between carbon (C = O) and nitrogen (N–H) groups [38,39]. The absorption bands around the amide I and amide II region in the infrared region have been utilized to assess and identify the secondary structure of proteins in different food matrices without the direct measurement using other methods or techniques [38,39]. The amide groups and other spectral regions associated with protein (e.g., N-H groups; C-H, O-H groups) have been correlated with the nutritional characteristics and structural characteristics of a sample [38,39,40]. Change in the secondary structure (α-helix and β-sheet) of proteins due to processing (e.g., extrusion, heating) or genetic modification is also possible by interpreting the MIR spectrum of the sample [38,39,40]. Other molecules such as polysaccharides (e.g., starch, pectin, glycogen) have a hydrogen bond that can be structurally linked with other polymers including cellulose microfibrils connecting with non-cellulosic polysaccharides (e.g., phenolic heteropolymer lignin), gel-like matrix of pectin linkage with other polysaccharides, and their cross-linkage with structural protein [41,42]. For example, the linkage between polysaccharides or between protein and lignin have been evaluated using vibrational spectroscopy [41,42,43].

### 4.2. The Analysis of Food and Human Interactions

The responses and the interactions between food and humans are of importance, even more so when the whole food matrix needs to be analyzed. One way to assess this response is through the utilization of sensory analysis. In this field, vibrational spectroscopy has been explored and reported as tool to predict sensory responses, and properties, as well as consumers’ responses to different food and beverage matrices [44]. Different authors and scientific reviews have reported the use of vibrational spectroscopy (e.g., NIR and MIR spectroscopy) to predict sensory properties and consumer responses in meat and fish [45], to measure olive oil sensory characteristics [46,47,48], to assess sensory properties in different types of tea [49,50,51] and coffee varieties [52,53,54] as well as the prediction of sensory characteristics in grape and wine samples [55,56,57]. Furthermore, these techniques have been evaluated to predict sensory attributes in cheese from different origins and types of milk [30,40,58]. These same techniques have also been explored for their ability to measure sensory properties in fruit samples such as apples [59], to assess the sweetness and flavour properties in oranges and grapefruit samples [60], to assess sensory traits in sweet potato [61], as well as the prediction of taste parameters in both peanuts [62] and peas [63].

Although NIR and MIR spectroscopy have been used in animal studies to predict digestibility in feeds, not many studies can be found that reported the use of these techniques to assess the digestibility and fermentability of foods in the field of human nutrition. Few studies have reported the use of NIR spectroscopy to predict the digestibility of either fibre components [64] or starch [65] in food. More recently, Ni and collaborators [66] and Ni and Cozzolino [67] presented and discussed different applications of vibrational spectroscopy to assess the interactions of food with humans via the analysis of biological fluids (e.g., saliva) and human tissues. These studies demonstrated the ability of vibrational spectroscopy to assess the interactions between food and humans.

### 4.3. The Definition and Utilization of Food Fingerprinting

**Figure 4:**
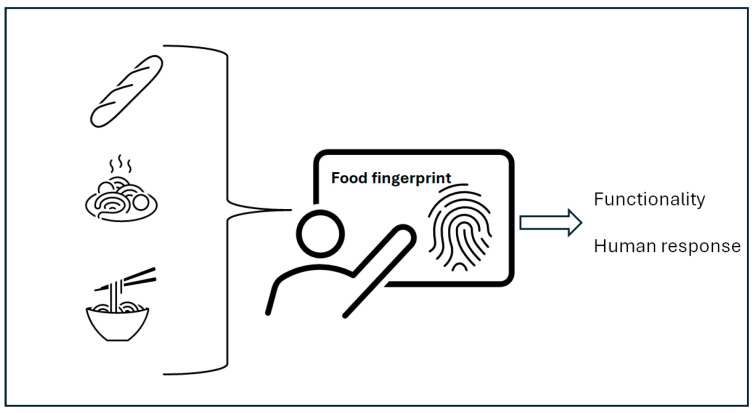
The utilization of food fingerprints based on vibrational spectroscopy as analytical tool to assess and understand food functionality and the interaction with human nutrition.

The fingerprint region (1500 to 450 cm^−1^) in the MIR range is defined as the region where the vibration from molecules is unique [28,68,69,70]. This unique characteristic in the spectrum provides information about the type and concentration of a given molecule present in a food. This allows for the location and definition of specific bonds (e.g., C-H, N-H, O-H) in the infrared region associated with carbohydrates, lipids or proteins. These bonds contribute to explaining the whole characteristic and properties of the food [28,68,69,70]. Furthermore, the spectral profile of the sample can be utilized to identify a single compound or more importantly to identify similarities (or dissimilarities) between foods. The fingerprinting also allows us to provide information about intermolecular interactions rather than the molecular structure. The utilization of the fingerprint region has been utilized to reveal the association between the molecular structure of the food, the nutritional value and digestive characteristics, as well as allowing to identify possible alteration of structure during processing [7,71] (see Figure 4). Examples on the utilization of food fingerprinting can be found in the work published by Munck and collaborators [72,73,74]. These authors used the NIR spectra to identify specific mutants in the endosperm of barley associated with (1→3, 1→4)-β-glucan [75]. The same authors reported the utilization of NIR spectroscopy to identify changes in lysine content in both the embryo and endosperm (e.g., different lysine content, high-lysine mutants) of barley mutants [74]. Bevin and collaborators [76] reported the ability of infrared to collect the fingerprinting of wine to monitor the integrity of wine samples during transport. Overall, the utilization of both infrared (IR) and Raman spectroscopy to record the so-called fingerprints is the basis of authenticity, fraud, contamination and safety applications in many food ingredients and products [77,78].

### 4.4. Modelling Storage and Shelf-Life of Food Ingredients and Products

The prediction of time of storage and shelf-life of different food ingredients and products has also been another field where vibrational spectroscopy has gained interest in recent years [79,80]. In these applications, it has been reported that the information contained in the spectrum was used to assess or to estimate the storage time of a food (e.g., meat shelf life), changes in the samples due to storage, or even to estimate the overall quality of the samples during storage [79,80]. These applications highlighted that the sole measurement of a single chemical compound does not provide information about the changes that occur during storage. Furthermore, it has been demonstrated that patterns or clusters in the data due to differences (or similarities) in the spectra can be more useful to monitor the changes associated with food storage or shelf-life [79,80,81,82,83,84,85].

### 4.5. Mapping the Chemical and Functional Properties of Food

The incorporation of NIR with hyperspectral (HSI) imaging is providing a new tool in food sciences [21]. NIR combined with HSI systems has the main advantage of integrating both spectroscopic and imaging techniques into one system, providing the ability to simultaneously collect spectral and spatial information [21,86,87,88]. This specific characteristic of the HSI system granted the collection of information from the different chemical components within a sample in addition to creating a map of the spatial distribution of these components within the sample [86,87,88,89]. These advantages provide us with the means to analyze the “food matrix” allowing us to show the distribution of chemical compounds and the interactions between them along the entire food matrix. This task can be achieved by the development of maps or images allowing us to interpret the differences or similarities between treatments or even recipes [86,87,88,89,90]. Examples on the ability of HSI to evaluate the processing method (e.g., sugar distribution) used to make Danish butter cookies [91], to assess the distribution of water in cassava after boiling [92], to monitor changes in meat nutritional properties after cooking [93] or the identification of the type of filing in sandwiches [94], have been reported. These examples have shown that it is possible to evaluate the whole food matrix, or to evaluate the optimal combination of ingredients in a food ingredient or product [21].

### 4.6. Monitoring and Traceability of the Food Manufacturing Process

Recently, process analytical technology (PAT) approaches have been incorporated to monitor and trace the processing of food ingredients, including fermentation monitoring [95,96,97]. The PAT approach has been developed to design, analyze and control the manufacturing process through timely measurements of critical quality and performance attributes with the objective of ensuring final product quality [95,96,97]. Consequently, the main objective of PAT is to provide real time analysis of the process. This approach helps to avoid or to minimize waste, to detect faults, and to improve efficiencies during the processing of food [98,99]. Vibrational spectroscopy (e.g., NIR, MIR) has been considered one of the most appropriate tools to obtain data or information to support decision management systems during the manufacturing of foods [98,99]. Overall, the PAT as well as other similar control systems based on vibrational spectroscopy are contributing to the efficient and reliable quality control of food processing. Furthermore, its implementation is allowing us to improve the traceability of the process, therefore assuring the consistency and integrity of the entire process along the food supply and value chain [98,99].

## 5. Considerations and Limitations—Research and Implementation

Although the utilization of vibrational spectroscopy as a tool in food analysis seems to be simple, there are several issues that need to be considered during the implementation of these techniques. The following sections describe some of the main issues that need to be considered before the utilization of these techniques.

### 5.1. Instrumentation

Vibrational spectroscopy (e.g., NIR, MIR, and Raman spectroscopy) has shown its potential to predict the chemical composition of food. However, its main benefits have been demonstrated with the analysis of the whole food sample (e.g., whole grain, intact meat) [25,26,100]. Regardless of its benefits, there are issues that are not well understood by the user during the application of these methods. It is well known that the spectrophotometers or instruments have differences (e.g., light source, wavelength range, instrument sensitivity and stability), even when they are from the same manufactures, due to their design. In addition, environmental factors either in the laboratory or outside the laboratory (e.g., dust, temperature and humidity) can influence the spectra collected [25,26,100]. Characteristics and properties of the sample analyzed (e.g., liquid, fresh, or dry sample) also influence the signal collected (e.g., spectrum) and consequently on the performance of the calibration (e.g., standard error of calibration, validation, and prediction) [25,26,100,101]. The specific characteristics or properties of the samples due to physiology or biology are also important variables to be considered during the evaluation of the results from a calibration or classification model.

### 5.2. Sampling

It is recognized that both data quality and sampling play an important role in model development (e.g., calibration and classification) [102,103,104,105]. Sampling error can dominate the analytical error by factors of five, ten or even higher, depending on various factors (e.g., sampling procedure, number of samples, remote sampling) [103]. Overlooking the effect of sampling has a critical effect on the analysis and interpretation of the data, and model or application [102,103,104]. Unfortunately, the definition of samples and method of sampling targeting the food matrix is not well understood. Furthermore, in many of the studies published, the standard error of the reference method used to develop the calibration model is not known or either unknown or not even reported [102,103,104].

Concomitantly with the sampling error, the analytical error is of high importance as this statistic is utilized to evaluate the ability of the calibration model, or in other words to evaluate the ability of a calibration to predict the same parameter [25,26,100,101]. The sampling method, or issues associated with the instrument such as signal to noise ratio (S/N), reporting spectra using different units (e.g., wavelengths, wavenumbers, frequency) are additional issues that contribute to the interpretation of the models [25,26,100,101]. The variability in both spectral characteristics and quality of the reference data, differences in the spectral region used, and the complexity of the model are influencing the accuracy and reliability of both calibration and prediction models.

### 5.3. Validation

Validation is of importance in any application of vibrational spectroscopy. Most of the applications reported and published have been validated using cross-validation or internal validation methods. The use of cross-validation determines challenges for the implementation of the models in real world applications where independent validation must be utilized to test the robustness of the models [106,107]. Different validation methods are available where various statistical parameters have been used to evaluate and report the performance of the models (e.g., calibration and classification) [104,105,106,107]. Some studies have also suggested that international collaboration will be required (e.g., development of protocols and guidelines, training) to improve the wide utilization of vibrational spectroscopy and to reduce time and cost of the validation [104,105,106,107].

**Figure 5:**
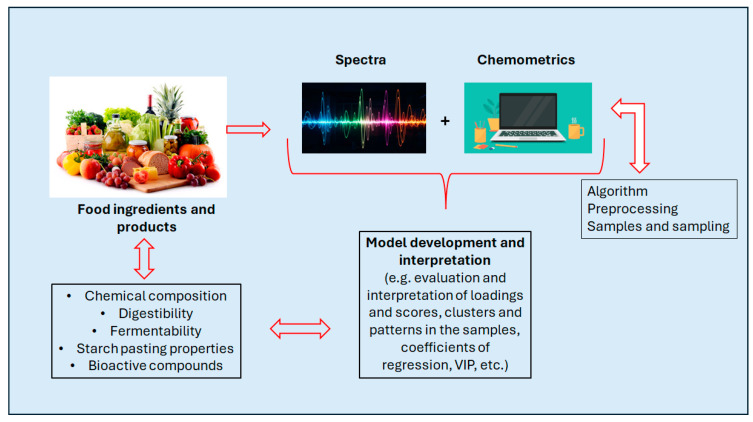
A schematic representation of the flow of relating spectra collection and data analysis to assess food functionality and nutritional value.

Figure 5 shows the flow of relating spectra collection and data analysis. This figure schematically explains how both functional and structural information can be extracted from spectrum, where the spectral information is interpreted using the patterns in the data set, loadings, coefficients of regression, etc.

## 6. Conclusions

This review showed the applications of vibrational spectroscopy (e.g., NIR and MIR spectroscopy) to assess the food matrix without the development of calibration models to predict the composition of food ingredients and products. The few studies published highlighted that the different vibrational spectroscopy techniques can analyze and provide information about the chemical, physical and functional characteristics of the food.

These applications showed that the spectrum of the food matrix can be analyzed using different algorithms and pre-processing techniques (e.g., derivatives, baseline) providing with a holistic view of the properties of food. Unfortunately, most of the examples have described feasibility studies, where small data sets were used to describe the food matrix.

The incorporation of vibrational spectroscopy to analyze the food matrix into routine analysis or practical application is facing different challenges. They include not only the technological and scientific challenges associated with the application and the interpretation of the data, but also other issues such as regulatory compliance, the integration of the predictive models into existing analytical frameworks, the requirement of friendly interface for end-users, data security and privacy issues, and their integration with ethical and welfare guidelines and legislation. Accordingly, protocols addressing these issues must be developed in collaboration with the user (e.g., industry) to ensure that the benefits of advanced data analysis are harmonized with the practicality and the specific requirements imposed by the end-user. The development of guidelines and international collaboration to develop large and robust datasets must be required to enable the use of models in routine conditions. Furthermore, training in human resources (e.g., industry, technicians, researchers and end-users) will be required to ensure the smooth applicability and interpretability of these tools in routine analysis of food (see Table 1).

## Figures and Tables

**Table 1:** Characteristics, advantages and limitations of different vibrational spectroscopy techniques.

	MIR	NIR	Raman	HSI
Wavelength range	4000–400 cm^−1^	12,500–4000 cm^−1^	2500–200 cm^−1^	MIR, NIR, Raman
Advantage/s	Fingerprinting and fundamental vibrations	Combination, in some cases fingerprinting	Water produces a weak Raman scattering	Spatial and spectra
Limitations	Samples with high moisture/water content. No possible to analyze whole or unground samples	Issues associated with limit of detection, low concentration of compounds	Weak signal, presence an intense fluorescence background noise	Data computing, complexity and time-consuming image analysis
Application/s	Homogenized or ground samples, liquid and pastes	Different types of samples (e.g., whole grain, feeds, plant parts and whole plant, liquids)	Analysis of samples with high water content	A wide range of sample types

MIR: mid infrared spectroscopy; NIR: near-infrared spectroscopy; HSI: hyperspectral imaging.

## Data Availability

No new data were created or analyzed in this study.
